# Sensory Perception and Food-Evoked Emotions of Older Adults Assessing Microwave-Processed Meals with Different Salt Concentrations

**DOI:** 10.3390/foods13040631

**Published:** 2024-02-19

**Authors:** Maria Laura Montero, Lisa M. Duizer, Carolyn F. Ross

**Affiliations:** 1 School of Food Science, Washington State University, Pullman, WA 99164, USA; marialaura.montero@ucr.ac.cr; 2 National Center for Food Science and Technology (CITA), University of Costa Rica, San José 11501-2060, Costa Rica; 3 Department of Food Science, University of Guelph, Guelph, ON N1G 2W1, Canada; lduizer@uoguelph.ca

**Keywords:** older adults, ready-to-eat-meals, microwave processing, sensory perception

## Abstract

This study employed a home-use test to explore the sensory perception and evoked emotions of older adults in the assessment of chicken pasta meals with different salt concentrations. Ready-to-eat (RTE) meals with three salt levels (100%, 75%, and 50%) and two treatments—with and without added herbs—were tested. Multiple sensory attributes and overall meal liking were evaluated by participants (*n* = 54; 60–86 years of age) with hedonic and just-about-right scores. Twenty-five food-evoked emotions were also tested. Sensory results suggested a 50% salt reduction is possible with minimal impact on the overall liking, while a 25% salt reduction did not affect the saltiness and flavor liking of the meals. Herb addition positively impacted the aroma, flavor, and spiciness liking of the meals. The emotions that differed (*p* < 0.05) among meals were active, aggressive, bored, calm, happy, and wild, with the meals with herbs added eliciting more positive emotions. A questionnaire elicited information about participants’ interest in healthy eating, food technology neophobia, and picky behaviors to determine the influence of these factors on participants’ salt consumption habits. Sensory acceptance data combined with questionnaires explored what influenced this group of older adults in their acceptance of and interest in RTE meals.

## 1. Introduction

Globally, the proportion of the population aged 60 years or older is increasing. In 2019, older adults numbered one billion worldwide [1]. This number is expected to increase to 2.1 billion by 2050 [2].

The natural aging process may detrimentally affect food choices, e.g., older adults tend to eat less and consume more energy-diluted grains [3]. Older adults are at constant risk of becoming malnourished. Malnutrition can be caused by factors such as health status (e.g., gastrointestinal issues), use of medication, education, and food neophobia, i.e., the reluctance to consume/avoidance of novel foods [4,5]. Food neophobia may also involve consumers’ reluctance to accept new technologies for food production; this phenomenon is known as food technology neophobia [6].

Ready-to-eat (RTE) meals are food products that have the potential to meet the multiple needs of older adults. RTE meals are pre-cleaned, pre-cooked, packaged, and ready for consumption without prior preparation or cooking beyond simple heating [7].

RTE meals can be processed by different means. One technology that has been reported to have less impact on the sensory and nutritional properties of multicomponent, highly viscous food products is microwave technology [8,9]. Within microwave technology (MT), microwave-assisted sterilization system (MATS) has multiple advantages compared to more traditional thermal processes. Sterilization processes like retorting often result in negative or undesirable effects on the food product being processed [9]. Meanwhile, with MT, the total heating time can be reduced three to five times compared to traditional processing techniques due to its ability to penetrate food and generate volumetric heat rather than rely only on thermal conduction [10]. For these reasons, MT is presumed to be a successful sterilization method that improves the shelf-life of products while the nutritional value and sensory properties that are critical for consumer acceptance (e.g., flavor) are not affected [9].

As people age, the decline in taste perception may have health consequences. A weaker perception of salty taste may induce older adults to season meals with excessive amounts of salt and therefore exceed the recommended salt intake [11]. Public health authorities around the world have highlighted the direct relationship between high sodium intake and the development of chronic diseases such as hypertension and kidney disease [12]. Therefore, the WHO recommends limiting the consumption of sodium to less than 2000 mg a day. By 2025, WHO Member States have agreed to reduce the salt intake in the global population by 30% [13].

The design and development of food products with low sodium content and without drastic changes in the sensory profile remain major challenges for the food industry [14]. One option to reduce the sodium content in food products is the use of flavor enhancers. This strategy allows for a decrease in sodium content without compromising the palatability and the intensity of the salty taste of food [15]. Herbs are promising saltiness enhancers [16]. Barnett et al. [17] determined that the addition of herbs to ready-to-eat (RTE) pasta meals enhanced the perception of saltiness at lower salt concentrations than at higher concentrations; thus, it was possible to reduce the salt content up to 50% without compromising the overall liking of the meals. In another study, Montero and Ross [18] investigated the effect of the addition of herbs on the saltiness perception of older adults (60–85 years of age) across different formulations of white sauce. The authors found that the addition of herbs compensated for salt reduction without a reduction in taste. In both studies, Barnett et al. [17] and Montero and Ross [18] used the same herbs: basil, black pepper, and garlic powder.

Given the expressed desire of consumers to eat well in a pleasant environment, the location where the product is assessed is also an important consideration in product development [19]. Very few studies have conducted sensory evaluations, such as home-use tests (HUTs), with older adults in more authentic environments. Svendsen et al. [20] evaluated the acceptance of meals developed for older adults in nursing homes in two different settings (outpatient and home settings) with different participants (>50 years). The authors achieved an authentic consumption setting by performing the sensory evaluation at the participants’ homes rather than in a sensory booth; however, liking results were not significantly influenced by the setting. Nevertheless, for products that are usually consumed in large portions or in a specific context, a HUT might be a better option to determine true consumer acceptance of a food product [21,22,23], such as RTE meals.

Another growing area in food product development is the measurement of food-evoked emotions. Craik and Salthouse [24] showed that the experience and regulation of emotions seem to be relatively well preserved in aging as compared to other cognitive functions. However, the few studies on the application of tools to measure food-evoked emotion have resulted in a limited understanding of the impact of age on those emotions [25]. Romaniw et al. [26] showed that, in older adults tested in a lab setting, positive evoked emotions were associated with higher liking scores, while negative evoked emotions were more related to disliking. These results did not differ from those found among younger adults.

The present study employed a HUT to explore the sensory perception and evoked emotions of older adults (>60 years) in the assessment of thermally sterilized chicken pasta RTE meals with different salt concentrations and with or without herb addition. This study also explored the application of a food choices questionnaire to characterize the group of participants and identify variables that may influence their salt consumption habits.

The hypotheses for this study were that participants’ sensory perception of the meals with herbs added and higher salt content would evoke more positive emotions. The variables that help to understand better the participants’ salt consumption habits are their picky eater status, as indicated by an inflexibility index, and their general health index score.

## 2. Materials and Methods

### 2.1. Participant Recruitment

The study protocol received the approval of the WSU Institutional Review Board for conducting tests with human subjects, under the title: Sensory evaluation and saltiness perception in older adults IRB # 17741-003.

Participant Recruitment and Selection: 54 participants were recruited through the WSU Sensory Evaluation Listserv, through snowball recruitment, and by email correspondence with community organizations. Most participants were staff or retirees of two universities in bordering states. Most were white (87%), ages 60 to 86. The sample was 65% female, aged 69.8 ± 7.3 years of age.

Some of the challenges faced during the recruitments were that the study was conducted during the peak of the COVID-19 lockdown in 2020 and was centered in a small, racially, and educationally homogenous rural college town. These factors resulted in a small number (*n* = 54) of highly educated older adults who were willing and able to participate in this exploratory study.

Three recruitment criteria included: participants had to be at least 60 years of age and living independently or unassisted; they could not have any known allergies to the ingredients in the meals; finally, they had to be available and committed to conducting the sensory testing from home at the three defined evaluation time points.

### 2.2. Meal Preparation and Materials

The procedure reported by Barnett et al. [17] was followed to prepare the meals. Most components of the meals were prepared the day before they were thermally sterilized by microwave-assisted thermal sterilization system (MATS). Descriptions of the meals and their abbreviations are presented in Table 1. Briefly, a total of six formulations were prepared utilizing three levels of salt content (100%—base formulation, 75%, and 50%) with and without the addition of herbs (0% or no herbs were added).

Materials: The sauce ingredients included deionized water (Milli-Q Reagent Water System, Millipore, Bedford, MA, USA), UHT whipping cream (Safeway brand, Pleasanton, CA, USA), unsalted butter (Darigold, Seattle, WA, USA), modified starch (THERMFLO, Ingredion, Westchester, IL, USA), salt (Morton, Chicago, IL, USA), basil leaves (McCormick, Hunt Valley, MD, USA), garlic powder (McCormick), coarse ground black pepper (McCormick), and salt-free southwest chipotle seasoning blend (Mrs. Dash, B&G Foods, Inc., Norwalk, CT, USA). Fettucine (DeCecco Fettucine no. 6; Fara San Martino, Italy), chicken breasts (Safeway brand, Pleasanton, CA, USA), sun-dried tomatoes (Bella Sun Luci, Chico, CA, USA), chopped frozen onions (Safeway brand), and UHT whipping cream (Safeway brand) were also included in the meals.

### 2.3. MATS Processing

MATS Processing: In total, 360 trays were thermally sterilized in a pilot-scale microwave-assisted thermal sterilization system (MATS) at Washington State University (WSU), Pullman, WA, USA. A detailed description of the system can be found in Tang [27] and Resureccion et al. [28]. Briefly, MATS is a closed system with four sections—preheating, microwave heating, holding, and cooling. Each section has its own water circulation system; each system has a pressurized tank and plate heat exchanger to control water flow, and the temperature can be pre-set. A pocketed mesh conveyor carries the food trays across the different sections of MATS [28]. The microwave heating section has four 915 MHz microwave single-mode cavities that are connected through a pressurized tunnel [27].

The specific processing conditions used to produce the chicken pasta meals are described by Barnett et al. [17]. In our study, all the meals were preheated at 61 °C for 35 min. The microwave heating time was 4.5 min, the holding time was 4.6 min, and the cooling time was 5 min. The move speed for the conveyor was 72.7 cm/min. The sterilization process was based on a processing schedule determined by preliminary trials. These trials identified conditions that would result in the process lethality (F_0_) value of greater than 6 min [17]. The processing of the 360 trays was performed within 4 days; 40 trays were processed per batch.

Each of the trays had a portion size of 300 g. The concentration of herbs was kept constant throughout the 6 formulations. In Table 2, the amount of ingredients is presented per meal or tray (in grams). However, the water, cream, butter, starch, salt, and all the spices (SW Chipotle, basil, pepper, and garlic powder) were all mixed together to prepare the sauce. We adjusted the amount of sauce added to each meal, so the total amount was 300 g. Hereafter, these trays are referred to as “meals”, and the term “pasta” refers to fettuccine pasta.

Following thermal processing, the total amount of trays (60 trays per formulation) was stored at 17.2 ± 0.5 °C. The meals were initially stored for 14 days until they reached salt equilibrium [17] and then while the sensory evaluation was conducted.

Microbial/Safety Testing: Meals were tested for safety prior to consumption. A total of 12 trays of each treatment were randomly selected and screened for the same type of microorganisms, as previously reported (Micro-Chem Laboratories (Seattle, WA, USA; [29]). Based on the microbial testing results, the chicken pasta meals were deemed safe for consumption at each of the evaluated time points.

### 2.4. Home-Use-Test (HUT)

Meal distribution: Two options were offered to obtain the chicken pasta meals: contactless pick-up or contactless drop-off. For contactless pick-up, the procedure followed the contactless curbside pickup of many grocery stores. For contactless drop-off, the procedure mimicked delivery protocols of grocery stores, with sample drop-off outside the participant’s home. On the evaluation day, the participants received an email with a link to the evaluation questionnaire.

Sample presentation: Randomized three-digit codes were assigned to identify each of the six meals tested. The order of presentation was randomized and balanced across the six formulations. To prevent fatigue during the sensory testing, the six meals were evaluated over a period of three weeks; thus, at each evaluation time point, each participant evaluated two meals. For the evaluation of the meals, the participants were instructed that they did not have to consume the entire meal but to taste enough of each component to conduct the evaluation. They were also instructed to take a 5 min break between the first meal and the second meal. Unsalted crackers (Nabisco, East Hanover, NJ, USA) and water bottles (235 mL, Kirkland Signature, Seattle, WA, USA) were provided as palate cleansers.

### 2.5. Questionnaires and Method

Evaluation questionnaire: On the first day of the HUT, the participants consented to their participation in the study and answered demographic questions. The questionnaire used to collect the participants’ responses was designed with Compusense^®^ at hand (Compusense, Inc., Guelph, ON, USA). Instructions to heat up the meals were provided to the participants on a sticker placed on the Ziploc bag that contained each meal. The instructions were the same as the ones reported by Montero et al. [29].

The questionnaire applied in this study contained different types of questions. A 9-point hedonic scale (1—dislike extremely to 9—like extremely) was used to evaluate the participants’ liking of the appearance, aroma, saltiness, taste, flavor, spiciness, texture of the pasta and the chicken, and their overall liking of the meals. For some specific attributes, just-about-right (JAR) questions were asked, ranging from 1 = not enough, 3 = Just about right, to 5 = too much. These questions were specifically about the saltiness, flavor, spiciness, firmness of the pasta, and texture of the chicken. Check-all-that-apply (CATA) questions were asked about taste, flavor, and texture attributes. The taste and flavor attributes were the following: salty, sour, sweet, bitter, umami, fresh, tomato flavor, artificial, bland, canned vegetables, herbal, dairy flavor, seasoning, and other. The texture attributes were juicy meat, dry meat, tough meat, moist meat, hard-to-swallow texture, creamy texture, grainy sauce, soft- noodles, firm noodles, slippery noodles, sticky noodles, and other. These attributes were selected based on previous work by Romaniw et al. [26].

Participants selected all the flavors, tastes, and texture-related attributes that they perceived while eating the meal. In the final section of the questionnaire, the participants answered the EsSense 25, a shortened version of the EsSense Profile^®^, which presents 25 emotional terms that are evaluated on a 5-point intensity scale (1 = not at all; 5 = extremely) [30]. The 25 terms were presented in alphabetical order [26,31].

Food choices questionnaire: As a follow-up to the HUT, a questionnaire composed of 63 questions related to food choices and salt consumption was applied. An overall description of the questionnaire is presented in Table 2. The following overall topics were included in the questionnaire: eating habits, general health interest (GHI), texture of the food consumed, food technology neophobia (FTN), consumption of RTE meals, and salt consumption habits. The questions were mostly taken from previous studies [32,33,34,35]. Specific questions were used to explore topics such as GHI, FTN, and the inflexibility index (IFI) (see Table 2); answers to these questions were used to determine different indices. The internal consistency of questions composed of more than two items evaluated using a Likert scale was determined with Cronbach’s alpha. Given the restrictions on sample size, the answers to these questions are presented as descriptive data (quartiles, median, mean, and range) as a way to augment the characterization of the sample.

The general health interest scale (GHI) measures general interest in healthy eating [35,36]. The participants answered 8 questions on a 7-point scale anchored at the extremes of 1 (strongly disagree) to 7 (strongly agree). For the interpretation of the score, the last four questions or negative statements were reversed. Each participant’s score was calculated as a sum of the scores given to each of the questions. The internal consistency of the items was satisfactory (Cronbach’s alpha = 0.874) [37].

The food technology neophobia scale (FTNS) was used to determine the score/level of neophobia in relation to food technology. The participants answered 13 questions on a 7-point scale anchored at the extremes of 1 (strongly disagree) to 7 (strongly agree). For the calculation of the FTN level, the scores of four questions were reversed so that higher scores corresponded to greater neophobia [38]. The individual scores were calculated as a sum of the scores given by each participant for each of the 13 items. The internal consistency of the items was satisfactory (Cronbach’s alpha = 0.645) [39].

The inflexibility index (IFI) was determined based on the responses to 12 questions that reflect the willingness to eat non-preferred foods that are presented or prepared in new ways [40]. The participants responded to these questions on a 7-point scale anchored at the extremes of 1 (strongly disagree) to 7 (strongly agree). The higher the score, the more likely the participant presented inflexible eating behaviors. The internal reliability of the items was satisfactory (Cronbach’s alpha = 0.817) [37].

**Table 2:** Description of the food choices questionnaire.

Topic	Variable(s) Measured	Question Structure and Description
Eating habits: Question 1 (Q1)–Question 8 (Q8)	Interest in healthy eating (8 items)	1 (strongly disagree) → 7 (strongly agree) Ex: I am very particular about the healthiness of food Maximum score of 56 [35]. Higher score indicates higher general health interest [34]
Texture of food consumed: Q9–Q10	Mouth behavior (2 items) [33]	Choose one that is most like you: Ex: I like foods that I can crunch/ Ex: I like foods that I can chew Participants classified into four categories: chewer, cruncher, sucker, or smoosher.
Food purchase drivers: Q11	Importance of different issues in food purchase [32]	1 (not important) → 5 (very important) Ex: Texture (of food)
Food technology neophobia: Q12–Q24	Food technology neophobia (13 items)	1 (strongly disagree) → 7 (strongly agree) Ex: New food technologies decrease the natural quality of food Maximum score is 91 [41]. The participants were divided into three groups that represented. Low: 46.4–53.5 Medium: 53.51–60.6 High: 60.61–91.0 The range corresponding to each group was defined from the average FTNS plus or minus one standard deviation [38]. Higher score indicates reluctance toward novel or new food technology
Inflexible eating behaviors: Q25–Q36	Inflexible eating behaviors (12 items). The 12 items are related to rigidity about how preferred foods are prepared and presented and refusal to eat non-preferred foods [40]	1 (strongly disagree) → 7 (strongly agree) Ex: If I dislike a food, I will not eat it under any circumstances Maximum score is 84 [40] Higher score indicates inflexibility
Consumption of RTE: Q37–Q45	Frequency of consumption	1 (never or rarely) → 7 (5 or more times per week). Participants that selected never or rarely (1) or 2–3 times per year were addressed to Q39 (Main reason for not consuming RTE meals)
	Main reason for not consuming RTE meals	Ex: Convenience-saves time
	Type of RTE meals mostly consumed Place where RTE meals are usually purchased	Ex: Unhealthy
	Time of consumption	Ex: Chilled-refrigerated Ex: Grocery store
	Location where RTE meals are usually consumed	Ex: At home, alone
	Time of preparation and consumption of RTE meals	Ex: Less than 10 min
	Frequency of checking different packaging information before deciding to purchase RTE meals [32]	1 (never) → 7 (always) Participants responded how often they consult each of the following packaging information before deciding to purchase RTE meals: Ex: Nutrition facts panel/salt content/
Salt use/consumption: Q46–Q49	Measurement of discretionary salt use (4 items)	1 (strongly disagree) → 7 (strongly agree) Ex: When I cook a home-cooked meal, I taste before adding salt Maximum score is 28 [34] Higher score indicates more salt used in daily situations
Personal norms related to salt consumption: Q50–Q53	Personal norms related to salt consumption (4 items) The items measure participants’ personal opinions of how they should act when choosing food products [34]	1 (strongly disagree) → 7 (strongly agree) Ex: I feel that is my duty to reduce my dietary salt intake Maximum score is 28 [34]. Higher score indicates more action toward reducing dietary salt intake
Awareness of the consequences of salt intake: Q54–Q63	Knowledge of salt-related diseases (10 items)	1 (strongly disagree) → 7 (strongly agree) Ex: “Salt in my food…”, Helps iodine uptake/ Maximum score is 70 [34] Higher score indicates increased knowledge of either positive or negative consequences of salt-related diseases

### 2.6. Data Analysis

XLSTAT 2022.4.1 (Addinsoft, New York, NY, USA) statistical software was used for all data analyses.

HUT Data: Two-way analysis of variance (ANOVA) at a 95% confidence level was conducted to evaluate the liking results of the different sensory modalities of the chicken pasta meals. The main factors were salt level (100, 75, and 50%), herb addition (no herbs added, herbs added), and panelist. Panelist was analyzed as a random effect. The interaction between salt level × herb addition was analyzed. Means were separated with Tukey’s HSD test.

For each of the meal formulations, the JAR scale results for flavor, saltiness, and texture of the chicken were interpreted with penalty analysis.

The CATA questions for the flavor taste and texture attributes were analyzed with Cochran’s Q test to identify significant differences among the meals for each of the sensory terms (α = 0.1) [42]. The results were presented in a correspondence analysis (CA) plot that was based on chi-square distances. Penalty analysis was also conducted on CATA responses to establish which attributes influenced overall liking of the meals (α = 0.05).

EsSense 25 data: A two-way ANOVA at a 95% confidence level evaluated the intensity of those emotions evoked during the consumption of the different chicken pasta meals. The main factors were salt level (100, 75, and 50%), herb addition (no herbs added, herbs added), and panelist. Panelist was analyzed as a random effect. The interaction between salt level × herb addition was analyzed. Means were separated with Tukey’s HSD test.

Food choices questionnaire: Answers to questions on this questionnaire formed the basis of indices to provide, given the limited sample size, more descriptive data for characterization of the sample. The selected indexes and questions included the GHI scale; the FTNS; the IFI; discretionary salt use questions; personal norms questions; questions about awareness of consequences of salt intake (positive and negative); and questions about the frequency of consulting the packaging information related to salt content before purchasing RTE meals. For the indices and the responses to the questions most related to salt consumption, the first quartile, median, and third quartile were calculated.

## 3. Results

### 3.1. Evaluation of the Liking of the Sensory Attributes of the Meals: Effect of the Level of Salt Content and Herb Addition

To evaluate the effect of salt content and herbs addition on the liking scores of the six meals, a two-way ANOVA was run. The salt content × herbs addition interaction was not significant for the liking scores of the evaluated attributes; therefore, the simple main effects were interpreted.

The influence of three levels of salt content on the liking of multiple sensory attributes of the chicken pasta meals was evaluated (Table 3).

The overall liking scores did not differ significantly (*p* = 0.13) among the three salt levels tested: 100% original formulation, 75% (thus 25% salt-reduced), and 50% (thus 50% salt-reduced) of the original formulation. The overall liking scores on the 9-point hedonic scale ranged between neither like or dislike (5) and like slightly (6). Based on the mean comparisons of the overall liking scores, it was possible to reduce the salt content of the meals by 50% without impacting the overall liking. A 25% salt reduction did not impact the liking of the saltiness and flavor of the meals.

In the analysis of the attribute of saltiness liking, the meals with no salt reduction (100) and the meals with the 25% reduction/75% salt content presented the highest liking scores. The meal with 50% salt concentration was significantly (*p* < 0.0001) less preferred compared to the other two meals.

The liking of the flavor of the meals was significantly (*p* = 0.007) impacted by the salt content. The mean liking score of the meal with no salt reduction (100) was significantly higher than the meal with the 50% salt reduction. However, the mean liking scores of flavor for the meal with 25% reduction/75% salt content did not differ compared to the scores for the other salt levels tested.

The liking of the spiciness was not significantly (*p* = 0.45) influenced by the salt content in the meals. Regarding the texture-related attributes, appearance, and aroma, no significant differences were observed in the liking scores across the three salt levels.

The mean scores of the overall liking of the meals were influenced (*p* = 0.02) by the addition of herbs (Table 4).

The mean score of overall liking ranged between neither like or dislike (5) and like slightly (6) on the 9-point hedonic scale, a finding parallel to that of the effect of the salt levels, with the same range and the same set of scale anchors.

The mean liking scores of multiple sensory attributes of the meals—the aroma, flavor, and spiciness—were significantly influenced by the effect of herb addition (Table 4). The liking scores of the aroma, flavor, and spiciness of the meal with herbs added were significantly higher than those from the meal with no herbs added. For the aroma, the liking mean score of the meals with herbs added was 6.4, which corresponds to a score between like slightly (6) and like moderately (7). For flavor, the liking mean score was 6.0, corresponding to like slightly (6). For spiciness, the liking mean score was 5.5, corresponding to a score between neither like or dislike (5) and like slightly (6).

However, for saltiness, the herb addition did not significantly (*p* = 0.08) increase the liking mean score of the meals. Furthermore, the liking mean score of the appearance did not present significant differences (*p* = 0.6) between the formulations with herbs added and those without herbs.

### 3.2. Effect of Some Taste–Flavor and Texture Attributes on the Overall Liking of the Meals

A list of taste–flavor and texture attributes was presented to the participants to better understand their sensory experience while eating the meals. Based on Cochran’s Q test (Table 5), out of 26 characterization attributes listed (14 for taste–flavor and 12 for texture), 11 were significantly different (*p* < 0.1) across the meals.

**Figure 1:**
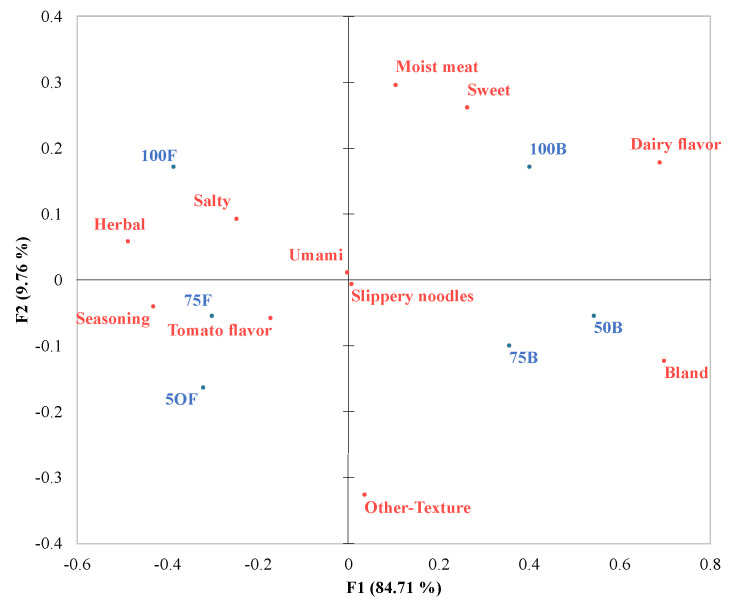
Correspondence analysis plot of the six different chicken pasta meals and the significant (*p* < 0.1) taste−flavor and texture attributes for CATA questions tested with older adults (*n* = 54).

A correspondence analysis plot was developed for those eleven taste–flavor and texture attributes that identify significant differences among the meals (Figure 1). Herb addition was the factor that most influenced the distribution of the meals in the plot space. The first two dimensions explained approximately 94.5% of the variance. F1, or the first dimension, explained most (84.71%) of the attributes and differentiation of the products. The meals with herbs added were described as herbal, salty, seasoning, and tomato flavor (Figure 1). Meal 100F was associated with a salty taste and herbal flavor. Meals 75F and 50F were rated similarly to each other and associated with the attributes seasoning and tomato flavor.

For each type of meal, meals with herbs and seasoning added are represented as F, and meals with no herbs nor seasoning added are represented as B. The salt content identified as 100% of salt content or the original formulation, 75% of the salt content of the original formulation, and 50% of the salt content of the original formulation are represented as 100, 75, and 50, respectively, following the meal name.

In contrast, the meals with no herbs added were described with attributes such as dairy flavor and bland and sorted into the positive values of the first dimension. Meal 100B was associated with attributes such as moist meat, sweet taste, and dairy flavor. Meals 75B and 50B were perceived to be similar to each other and associated with bland flavor.

The other attributes—umami taste, slippery noodles, and other texture—were not clearly associated with a specific meal. For the term other, terms such as oily and stringy/chewy meat were those most frequently mentioned by the participants.

**Figure 2:**
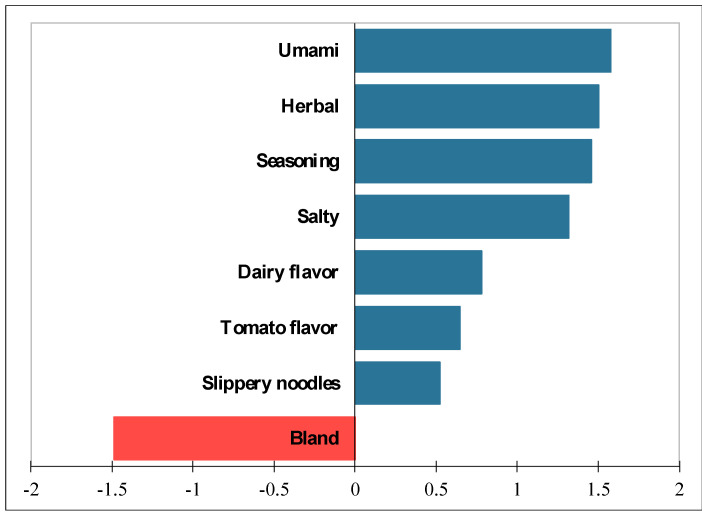
Mean impact of some taste–flavor and texture attributes evaluated in terms of the overall liking of six RTE meals processed with MW technology and evaluated by older adults (*n* = 54).

We also analyzed the influence of the attributes that differed significantly (*p* < 0.1) with the overall liking of the meals. The attribute of bland flavor provoked a significant drop (*p* < 0.0001) of 1.5 in the overall liking scores of the meals (Figure 2). Bland was selected as an attribute present in the meals 40% of the time. Umami and salty tastes, as well as herbal and seasoning flavors, had the opposite effect; the presence of these attributes resulted in significant increases (*p* < 0001) in the overall liking: 1.58, 1.32; 1.50, and 1.46 units, respectively. Umami taste was ticked 42% of the time and seasoning 52% of the time as attributes present in the meals.

### 3.3. Improvement in Sensory Characteristics

Saltiness intensity was penalized by the participants, mostly for the meals with 75% and 50% salt concentrations and no herbs added. For those meals, 56% and 72% of the participants perceived the meals as not salty enough. 75F and 50F comprised close to 50% of the mentions on the JAR anchor. For example, 50F was considered not salty enough by 48% of the participants and 50B by 72% of the participants. For 50F, the perception of not salty enough was associated with a significant mean drop (*p* = 0.04) of 1.2; for 50B, this perception was associated with a significant mean drop (*p* = 0.004) of 1.8 units.

The flavor of the meals was also evaluated with JAR scales. As with saltiness, the meals with herbs added presented more responses at the JAR point than those with no herbs added.

### 3.4. Food-Evoked Emotions in Meal Consumption: Effect of Salt Level Content and Herb Addition

Of the 25 emotions evaluated, the ratings for the food-evoked emotions good, interested, understanding, and warm significantly differed (*p* < 0.05) among the meals due to the effect of the salt level (Table 6). This set of food-evoked emotions is mostly associated with positive emotions, except for understanding, which is not clearly binary [31].

Overall, the ratings of those food-evoked emotions that differed significantly due to salt level in the meals ranged between slightly (2) and moderately (3) along the 5-point scale.

The positive emotion good received a mean score of 2.8 and was significantly (*p* = 0.001) more intense in the meals with no salt reduction (100) when compared to the rating mean score (2.4) obtained for the meals with 50% salt reduction (50). However, the rating mean score (2.6) of the meals with 75% salt content (25% reduction) did not differ from the means obtained for the meals with either no salt reduction (100) or 50% salt content (50). This trend was also observed for understanding. These results aligned with the hedonic evaluation; the meals with 75% salt content (25% salt reduction) received ratings similar to the original formulation (100).

For the meals with the three different salt level contents, the scores of interested, another positive food-evoked emotion [31], ranged between 2.5 and 2.7. The meals with 75% salt content evoked a significantly higher (*p* = 0.03) rating mean score than the meals with 50% salt content. Nonetheless, the mean score (2.7) of the meals with 75% salt content did not differ from the mean (2.6) for the meals with the original formulation (100).

The emotion warm evoked a significantly (*p* = 0.006) lower rating score for the meals with 50% salt reduction when compared to the rating mean score of the meals with the other two tested salt levels. There were no significant differences between the mean scores of the meals with 100% and 75% salt content.

The effect of herb addition significantly impacted the mean scores of five different food-evoked emotions: active, aggressive, bored, calm, happy, and wild (Table 7). Most of these food-evoked emotions are categorized as positive, except for aggressive (unclear classification) and bored (negative classification) [31].

Overall, the ratings of the food-evoked emotions that differed significantly due to herb addition in the meals ranged across not at all (1), slightly (2), and moderately (3) along the 5-point scale.

For active, happy, and wild, three positive food-evoked emotions, and aggressive (unclear classification), the mean scores of the meals with herbs added (F) were significantly higher (*p* < 0.05) than the mean scores of the meals with no herbs added (B).

*Calm* was rated significantly higher (*p* = 0.002) in the meals with no herbs added. The same trend was observed for bored, the only negative food-evoked emotion that differed significantly (*p* < 0.0001) between the two treatments (herbs vs. no herbs added).

### 3.5. Food Choices Questionnaire

As shown in Table 8, the mean value for GHI was 36 out of a maximum score of 56. The scores ranged from 19 to 54. The first (Q1) and third (Q3) quartiles were determined to look for segmentation of the participants based on their responses to some of the questions on the food choices questionnaire. The estimation of Q1 showed that 25% of the participants’ responses were below 30 and 75% were below 42 (Q3).

In terms of food technology neophobia (FTN; Table 8), the participants were categorized into three different groups: low, medium, and high levels of FTN. The low level ranged between 46.4 and 53.5, the medium level between 53.5 and 60.6, and the high level between 60.6 and 91.0. A total of 40.8% of the participants showed a low level of FTN (neophiliacs), 34.7% showed a medium level, and 14.3% showed a high level (neophobics). The FTN scores for the participants ranged between 39.0 and 70.0. Q1 indicated that 25% of the participants’ responses were below 49 (low level of FTN); meanwhile, Q3 showed that 75% of the participants’ responses were below 57.0 (medium level of FTN). These descriptors showed that the participants are not particularly reluctant to try food products processed with new technologies.

Another variable that was measured was the IFI of each participant. In our study, the mean value (30.7) of the IFI indicated that overall, the participants did not tend toward picky behaviors. The scores obtained by the participants for IFI ranged between 15.0 and 56.0. The values for this index may range between 12 and 84 [40]. The first quartile showed that 25% of the participants’ responses were below 23.0, while Q3 showed that 75% of the participants’ responses were below 36.0. These results indicated that from the set of 12 questions, the participants most frequently expressed somewhat disagree with statements such as I avoid letting different foods touch my plate, even when they are both foods that I like; I do not like mixed foods (like stews or casseroles or mixed vegetables).

Because the main objective of the present study was to determine the acceptance of RTE meals with reduced salt content, we included questions related to salt intake that had been previously used. These questions were related to discretionary salt use and reflected the degree to which salt is added to daily eating. These results indicated that the participants most frequently expressed to be neutral about statements such as when I cook a home-cooked meal, I taste it before adding salt; when I eat my home-cooked food, I add salt at the table. The results overall showed the participants did not move much toward salt addition before or when cooking or eating food.

Another subset of questions related to personal norms (how respondents react emotionally when choosing dietary salt) was also part of the survey. Of these questions, 53% of the participants scored higher than the mean (12.5, see Table 8). Q3 indicated that 75% of the responses were below a value of 15. The minimum and maximum scores obtained from the participants’ responses were 4 and 19, similar to the range reported by Mørk et al. [34].

Overall, the participants were aware of the positive and negative consequences of salt intake but relatively uncommitted to taking explicit salt-reducing action. The mean score for their knowledge of negative consequences was slightly higher than their mean for knowledge of the possible benefits of salt. A total of 75% of the participants’ responses were under 20.0 in terms of the awareness of both positive and negative consequences associated with salt consumption. For the set of questions related to the positive consequences of salt intake, the participants’ responses ranged between 9.0 and 22.0, and the responses associated with the negative consequences ranged between 14.0 and 25. According to Mørk et al. [34], the score for each set of questions (positive and negative consequences, respectively) ranged from 5 to 25. The Q3 values point to the participants of the study having sufficient/adequate knowledge or awareness of the positive and negative impact of salt consumption in their diet.

Another variable used to characterize the participants of the study was their preferred oral processing behavior. Most participants self-identified as chewers (46.9%) and crunchers (42.9%), 10.2% as smooshers, and none as suckers.

The questionnaire also investigated the participants’ frequency of consumption of RTE meals. The frequency of 44.9% of the participants ranged from every 2 to 3 weeks to 5 or more times per week. Participants in this range indicated that some of their reasons for consuming RTE meals were convenience/saves time (36.7%) or convenience/saves physical and/or mental energy. The participants also indicated that the type of RTE meals they most often consumed were frozen and chilled/refrigerated. Participants most often consumed frozen and chilled/refrigerated RTEs, and mostly at dinner at home with other family members.

## 4. Discussion

### 4.1. Evaluation of the Liking of the Sensory Attributes of Meals: Effect of the Level of Salt Content and Herb Addition

This exploratory home-use study determined that older adults living independently rated the liking of the saltiness and flavor of the meals differently. However, in terms of overall liking, it was possible to reduce the salt content of the meals by 50% without impacting this important attribute. The mean liking scores were affected by the salt level content of the meals tested. For these attributes, the 25% salt reduction was equally preferred to the 100% formulation. These results align with previous research performed in an in-lab setting that showed that it was possible to reduce the salt content of the meals by 25% without compromising the overall liking [26]. However, Romaniw et al.’s [26] study generated overall higher liking scores, which may be attributable to the demographics (a larger sample size) and the environments in which the tests were run (in lab vs. at home).

Herbs and spices have been widely used as flavor enhancers of food and, more recently, have become an effective strategy for salt reduction in food [17,43]. This strategy has been investigated in different food products (e.g., soups, mashed potatoes, baked chicken, sauces, and RTE meals) [17,18,43,44,45]. In older adults, the flavor enhancement strategy is primarily used to improve their nutritional value [43]. Herb and spice addition have been successfully used to increase the saltiness intensity of dishes (tomato soup, mashed potatoes, and baked chicken) to the level of control with no salt reduction [43]. In the present study, the herb addition positively impacted (*p* < 0.05) the mean of the overall liking and the liking scores of aroma, flavor, and spiciness of the thermally processed meals. However, herb addition did not impact the mean liking scores of saltiness of the meals, unlike the findings reported by Tomić-Obrdalj et al. [43].

### 4.2. Effect of Taste–Flavor and Texture Attributes on the Overall Liking of the Meals

To determine the effect of multiple taste–flavor and texture attributes on the overall liking of the meals (as measured by CATA questions), the interactive effect of salt level and herb addition were analyzed. From the analysis of the CATA questions, the taste–flavor attributes (e.g., umami taste, herbal, seasoning) primarily characterized the meals.

In the present study, the main driver of dislike of the meals was bland. It has been recently reported that in food products such as RTE meals, there is a growing demand for seasoning ingredients to provide potential functionality and diverse flavor [46]. Supportive research shows that in a group of older adults (*n* = 100), the addition of spices (e.g., black pepper, garlic powder, onion powder, ground mustard, and dill) significantly increased the overall liking and the flavor intensity of vegetarian and meat-based entrées and meals [47]. The liking of certain herbs or spices could have influenced the participants’ perception of the meals.

### 4.3. Improvement in Sensory Characteristics

The addition of herbs to the meals enhanced the detection of saltiness. In meals with the same salt concentration, the added herbs positively impacted the enhancement of salty and the detection of saltiness, a finding that points to herbs as an effective strategy in salt reduction [16,17,26].

Salt reduction is an elusive product development goal complicated by evidence that salt is the basic taste with the most evident decline in intensity perception in the aging process [48]. The consequence can be oversalting, a habit some try to avoid. Older adults who voluntarily consume less salt in their diet could be more sensitive to salt reductions or differences in salt content than those who have a high-salt intake diet as a result of diminished ability to detect salt [49]. The participants of our study generally appeared to have been sensitive to the salt reduction in these meals.

Some more specific attributes related to flavor—umami taste, herbal, and seasoning—had a significant positive impact on the overall liking of the meals; those terms were mentioned as present in or characteristic of the meals. This finding may explain why the antonymous term bland is associated with a negative effect on the overall liking of the meals. This association points to the potential use of herbs in improving the flavor of a product with reduced salt content.

### 4.4. Food-Evoked Emotions in Meal Consumption: Effect of Salt Level Content and Effect of Herb Addition

Multiple studies have reported the benefits of evaluating food-evoked emotions during food consumption as a mechanism to understand better food acceptance and predict food preferences in consumers [50,51,52]. In our exploratory study, positive emotions were mostly evoked in the participants when the salt level of the meals varied. These emotions were evoked at a higher intensity, mostly for the meals with the original formulation and those with 75% salt content (25% salt reduction).

The scores for positive emotions were usually higher for those meals with herbs and chipotle seasoning added, an outcome that supports the observed results from the hedonic evaluation and JAR results. Another aspect that could have influenced the more positive responses to the meals with herbs added is that individuals who are high in the trait of sensation seeking tend to like spicy foods more than individuals low in that trait [53].

A finding that points to how the salt reduction may have evoked the positive emotion good was that this emotion was evoked at a significantly higher level for the 100 meals (original formulation) than for the 50 meals (50% salt reduction). The meals with 75% salt content had more of a middle point in evoking the emotion good among the participants of the study.

The emotion interested, associated with a positive arousal, was mostly associated with the meals with 75% salt level content. Deactivation and displeasure have been related to diminished flavor intensity [54]; this could explain why the meals with 50% salt reduction evoked the lowest intensity of this emotion in our group of older adults. These meals (50) also obtained the lowest mean liking scores for flavor liking when compared to the original formulation (100%).

In a consumer study (*n* = 100, 35.4 ± 11.1 years old), food matrices, including herbal infusions, evoked emotions such as joyful, active, and understanding. These sets of emotions are related to affection, liveliness, and understanding [55]. In our case, the emotions evoked by the meals also included understanding, and this emotion was evoked in combination with other positive emotions such as the ones discussed (good, interested, and warm).

The positive emotion warm was linked with the meals with the two higher salt content levels. This finding could indicate or reinforce a preference by the participants of the study toward these two types of meals as compared to the ones with 50% salt reduction.

In our exploratory study, the herb addition enhanced the strength of the food-evoked emotions such as active. This particular emotion has been associated with high arousal or activation. Meanwhile, the emotion calm has been associated with pleasurable or positive deactivation, and the emotion bored has been associated with unpleasant or negative deactivation [54]. Even though the emotion aggressive has an unclear classification, it could relate to the emotions previously mentioned. The herbs and chipotle seasoning added to the meals seemed to cause activation in the participants, an outcome that supports the observed results from the hedonic evaluation and JAR results. Similar results were reported by Romaniw et al. [26] regarding the evoking of the same type of emotions when the meals were evaluated in an in-lab setting by Canadian seniors.

Meanwhile, the participants of our study associated the emotion calm, a generally positive descriptor that indicates a pleasurable deactivation (e.g., peace, relaxed) [31,54,55], with meals with no herbs added. These meals could have been perceived as more simple or bland. Jaeger et al. [54] reported that the perception of lower flavor intensities was linked to deactivation to displeasure. This could be linked to our findings of the meals with no added herbs that evoked a bored emotion. Participants may have been bored with the lack of flavor in the meals. Romaniw et al. [26] also reported that bored had a negative connotation, like displeasure, and deactivation with no energy/intensity.

### 4.5. Food Choices Questionnaire

The mean value of the GHI indicates that the participants presented a medium level of interest toward healthy eating. These findings contribute to a better understanding of how the group of participants of the study are interested, value, or have intentions for this practice [34]. Roininen et al. [35] studied the use of the GHI in a group of Finnish adults (*n* = 1005) between the ages of 18 and 80 years. Some of the authors’ main findings indicated that age and gender affected the responses. Older respondents were more concerned with health as compared to younger respondents. This aligns with the findings of our exploratory study and reinforces the importance of looking into the health interests of this population.

In terms of food technology neophobia, about 75% of the participants showed a low-to-medium level of this neophobia. A study conducted by Soucier et al. [5] classified a group of 250 older adults according to their food neophobia level as 34.4% low, 35.2% medium, and 34.4% as high. Although our study and Soucier’s measured different but topically related constructs (food vs. food technology), the general breakouts of data were similar. Soucier et al. [5] also reported that the food neophobia scores were significantly higher for the participants who were very or somewhat unwilling to try new food products. This finding highlights the importance of exploring food neophobia in older adults as a possible driver of restricted food choices in this population. A review of the literature has not located any other research on the construct of food technology neophobia in older adults.

Another variable that may influence older adults’ food choices or compromise their diet quality is pickiness or inflexible eating behavior. The IFI reflects the willingness to eat non-preferred foods or preferred foods that are prepared or presented in new ways [40]. Zickgraf and Schepps [40] reported in a group of adults (18 years of age and over) a mean score of 27.7 and a maximum score of 60 on a 5-point scale that measured pickiness. That mean score aligns with 38.8 on the 7-point scale of the IFI. In our exploratory study, the scores of 26.5% of the participants on the IFI were equal to or higher than 38.80, an indication that this fraction of the participants was more likely to be picky eaters or have inflexible eating behavior. On the other hand, Zickgraf and Schepps [40] reported a mean of 10.21 for non-picky eaters on their 5-point scale. On a 7-point scale, that value equals 15; only 12.2% of the participants in our study obtained a score of 15 or close to 15. The implications of pickiness were explored by Maitre et al. [56], who suggested that food selectivity in older adults may predispose them to malnutrition from a limited dietary variety.

Discretionary salt use, a measure developed by Mørk et al. [34], indicates how respondents may take more action on a daily basis toward reducing dietary salt intake. The results showed the participants did not have a high discretionary salt intake. This finding could explain why the overall liking of the meals studied was mostly influenced by the salt level content.

Personal norms also help explain the role of salt in food choices. The higher the score for this set of questions, the more the respondent agrees with feeling bad, experiencing a bad conscience, or feeling that they should choose to reduce their dietary salt intake [34]. Our findings showed that the participants were satisfied and relatively unperturbed by not reducing salt, as evidenced by replies to questions such as the degree to which they feel bad when they are not deducting their dietary intake or feel that it is their duty to reduce their dietary salt intake. An indicator of a well-informed group of participants was their awareness of the positive and negative consequences of salt intake.

Regarding the texture behavior of the participants, almost half of the participants self-identified as chewers and over 40% as crunchers. Smoosher was the mouth behavior not selected by any participants. In a study conducted by Kim and Vickers [57], the authors categorized over 200 participants (age range not indicated) into the same four mouth-behavior groups. A total of 48% of the individuals self-identified as chewers, 41% as crunchers, fewer than 10% as smooshers (7%), and fewer than 5% as suckers. Overall, the results of their study align with our findings. Research has shown that mouth behavior drives food texture choice and preferences [58]. Considering the participants of our study were mostly chewers and crunchers, that could help to explain the fact that the texture of the chicken was attributed the lowest liking scores. In a study conducted by Jeltema et al. [58], participants categorized as crunchers and chewers showed lower preference for food products that were “very hard” or foods that could not be crunched or chewed.

Determining the frequency of the consumption of a food product can become a key factor during the process of developing new food products. The last set of questions of the food choices questionnaire explored the frequency of consumption of RTE meals. The participants’ main driver for the consumption of frozen and chilled/refrigerated meals was convenience. This characteristic is one of the main benefits of RTE meals.

The food choices questionnaire served as a useful tool to explore different characteristics, practices, and knowledge of the participants toward health, picky eating, salt intake, texture perception, and consumption of RTE meals. This questionnaire has the potential to be further used with a larger sample size to understand consumer segmentation.

### 4.6. Limitations and Future Studies

This exploratory study broke new ground in using a HUT with older adults for a period of three weeks during a time of contact restrictions during the COVID-19 pandemic. The focus on ways to reduce salt in the meals of older adults with the use of herbs to compensate for salt reduction is also innovative. The sensory evaluation of food-evoked emotions, an often-overlooked part of food product development, was novel as was the inclusion of the food choices questionnaire to complement the HUT was also innovative.

At the specific time when the HUT was conducted, December 2020, staying at home was promoted as a way to stay healthy and prevent COVID-19′s spread. This study was conducted in a region that borders two states. The access to older adults (60 years or older) living independently was limited, considering this population was at high risk and represented less than 10% of the demographic in the Pullman (WA) area.

Limitations of our study and recommendations for future studies include the following factors. The reduced number of participants may have limited obtaining more significant differences in the liking scores of the evaluated sensory attributes of the meals. Therefore, future studies should increase the number of participants (80–100) to contribute to more detailed discrimination of the multiple sensory attributes evaluated in the meals, particularly the overall liking and the liking of saltiness and flavor.

Conducting both an in-lab and home-use test with older adults might aid in determining the potential effect of the environment on the acceptance of the meals. Thus, future investigations could evaluate the use of other salt concentrations, herb types, and content. Other studies could examine the interaction of food neophobia and food technology neophobia to determine the extent to which these variables influence the food choices of older adults. CATA questions determine the strength of food-evoked emotions because this questioning technique is perceived as less difficult to relate to and more relevant than a scale [59]. Temporal dominance techniques (e.g., concept temporal dominance of emotions (TDE)) to measure food-evoked emotions are also recommended. It is also suggested to include questions related to the similarity of RTE meals or other food products addressed to older adults in homemade products. These types of questions may allow us to explore the impact of nostalgia on the acceptance or liking of food products in this population.

## 5. Conclusions

This study implemented different methodologies to explore the sensory perception and evoked emotions of older adults (>60 years) in the assessment of thermally sterilized chicken pasta meals with different salt concentrations and herb additions. The liking of some of the sensory attributes evaluated (saltiness and flavor liking) was impacted by the salt level content. The herb addition significantly impacted the liking of the aroma, flavor, and spiciness. Overall, the meals with herbs added were characterized by more positive descriptors (e.g., seasoning, tomato flavor, herbal, salty). The food-evoked emotions assessment provided a better understanding of the perception of the meals by older adults; the salt reduction and the herb addition evoked mostly positive emotions as compared to negative ones. The use of food-evoked emotions contributed to determining differences across the tested meals.

The application of a food choices questionnaire as a follow-up allowed for a more comprehensive characterization of the participants of the study in order to determine some common practices associated with lifestyle, eating behaviors, and salt consumption.

This exploratory study suggests that it is possible to reduce the salt content of meals with herbs added by up to 50% without impacting the overall liking and up to 25% without impacting the saltiness and flavor liking. The application of herbs in the development of RTE meals with reduced salt content targeted at older adults is potentially useful as a public health measure. However, improvement in texture-related attributes is also highly recommended for meat ingredients.

## Figures and Tables

**Table 1:** Chicken pasta meal labeling and formulations with and without herb addition (adapted from Barnett et al., [17]).

Label ^1^	Ingredients (g) ^2^				Sauce Ingredients (g)								
	Pasta	Chicken	Tomato	Onion	Water	UHT Cream	Butter	Starch	Salt	SW Chipotle	Basil	Pepper	Garlic
100F	78	76.5	5.5	2.2	68	62	2.1	1.5	1.08	2.1	0.2	0.2	0.3
75F	78	76.5	5.5	2.2	68	62	2.1	1.5	0.81	2.1	0.2	0.2	0.3
50F	78	76.5	5.5	2.2	68	62	2.1	1.5	0.54	2.1	0.2	0.2	0.3
100B	78	76.5	5.5	2.2	68	62	2.1	1.5	1.08	0	0	0	0
75B	78	76.5	5.5	2.2	68	62	2.1	1.5	0.81	0	0	0	0
50B	78	76.5	5.5	2.2	68	62	2.1	1.5	0.54	0	0	0	0

^1^ For each type of meal, meals with herbs and seasoning added are represented as F, and meals with no herbs nor seasoning added are represented as B. The salt content identified as 100% of salt content of the original formulation, 75% of salt content of the original formulation, and 50% of salt content of the original formulation are represented as 100, 75, and 50, following the meal name. ^2^ Weights are per 300 g tray.

**Table 3:** Effect of the salt content on the mean liking scores of the sensory attributes evaluated by a group of older adults (*n* = 54) in a set of six thermally sterilized chicken pasta meals with different salt concentrations.

Salt Level	Sensory Attribute							
	Appearance	Aroma	Saltiness	Flavor	Spiciness	Texture of the Pasta	Texture of the Chicken	Overall Liking
100 ^1^	5.5 ± 2.0 a ^2^	6.0 ± 1.8 a	5.6 ± 1.8 a	6.1± 1.8 a	5.2 ± 2.1 a	5.7 ± 2.0 a	5.0 ± 2.0 a	5.4 ± 1.9 a
75	5.4 ± 1.9 a	6.3 ± 1.8 a	5.2 ± 1.9 a	5.8 ± 2.0 ab	5.0 ± 2.3 a	5.4 ± 2.2 a	4.8 ± 2.2 a	5.2 ± 2.2 a
50	5.6 ± 1.9 a	6.2 ± 1.7 a	4.6 ± 1.9 b	5.5 ± 2.1 b	4.9 ± 2.1 a	5.4 ± 2.1 a	4.6 ± 2.0 a	5.0 ± 2.1 a
*p*-value	*p* = 0.66	*p* = 0.15	*p* < 0.0001	*p* = 0.007	*p* = 0.45	*p* = 0.14	*p* = 0.16	*p* = 0.13

^1^ For each type of meal, the salt content identified as 100% of salt content or the original formulation, 75% of salt content of the original formulation, and 50% of salt content of the original formulation are represented as 100, 75, and 50, respectively. ^2^ Results are presented as the liking means scores ± S.D. Different letters within a column (a, b) indicate that the tested sensory attribute mean value was different among the meals (*p* < 0.05), as determined by using Tukey’s HSD on a 9-point hedonic scale.

**Table 4:** Effect of the herb addition on the liking mean scores of the sensory attributes evaluated by a group of older adults (*n* = 54) in a set of six thermally sterilized chicken pasta meals with different salt concentrations.

Herbs Addition	Sensory Attribute							
	Appearance	Aroma	Saltiness	Flavor	Spiciness	Texture of the Pasta	Texture of the Chicken	Overall Liking
F (herbs added) ^1^	5.6 ± 2.0 a ^2^	6.4 ± 1.7 a	5.3 ± 1.9 a	6.0 ± 1.9 a	5.5 ± 2.2 a	5.6 ± 2.1 a	4.9 ± 2.0 a	5.4 ± 2.1 a
B (no herbs)	5.5 ± 1.9 a	5.9 ± 1.8 b	5.0 ± 1.9 a	5.5 ± 2.0 b	4.6 ± 2.0 b	5.4 ± 2.1 a	4.6 ± 2.1 a	5.0 ± 2.0 b
*p*-value	*p* = 0.48	*p* = 0.002	*p* = 0.08	*p* = 0.005	*p* < 0.0001	*p* = 0.22	*p* = 0.07	*p* = 0.02

^1^ For each type of meal, meals with herbs and seasoning added are represented as F; meals with no herbs nor seasoning added are represented as B. The data for the mean estimation were collapsed along salt level. ^2^ Results are presented as the liking means scores ± S.D. Different letters within a column (a, b) indicate that the tested sensory attribute mean value was different among the meals with or without herbs added (*p* < 0.05), as determined by using Tukey’s HSD on a 9-point hedonic scale.

**Table 5:** Cochran’s Q test (α = 0.1) identifies significant differences among the meals for each of the sensory taste–flavor and texture terms presented in the CATA questions.

Attributes	*p*-Values
Texture-related	
Moist meat	0.056
Slippery noodles	0.042
Other texture	0.042
Taste and flavor related	
Tomato flavor	0.000
Sweet	0.069
Bland	<0.0001
Herbal	<0.0001
Salty	0.001
Dairy flavor	<0.0001
Umami	0.085
Seasoning	<0.0001

**Table 6:** Effect of the salt content on the mean scores of the significant food-evoked emotions as rated by a group of older adults (*n* = 54) in a set of six thermally sterilized chicken pasta meals with different salt concentrations.

Salt Level	Food-Evoked Emotions				
	Adventurous	Good	Interested	Understanding	Warm
100 ^1^	1.9 ± 1.0 a ^2^	2.8 ± 1.1 a	2.6 ± 1.0 ab	2.3 ± 1.2 a	2.7 ± 1.2 a
75	1.6 ± 0.9 b	2.6 ± 1.1 ab	2.7 ± 1.0 a	2.2 ± 1.2 ab	2.7 ± 1.2 a
50	1.0 ± 0.0 c	2.4 ± 1.1 b	2.5 ± 1.0 b	2.0 ± 1.1 b	2.4 ± 1.2 b
*p*-value	*p* < 0.0001	*p* = 0.001	*p* = 0.03	*p* = 0.02	*p* = 0.006

^1^ For each type of meal, meals with the salt content identified as 100% of salt content or the original formulation, 75% of salt content of the original formulation, and 50% of salt content of the original formulation are represented as 100, 75, and 50, respectively, following the meal name. ^2^ Results are presented as the means scores ± S.D. Different letters within a column (a, b, c) indicate that the tested emotion mean value was different among the meals (*p* < 0.05), as determined by using Tukey’s HSD on a 5-point scale (1 = not at all; 3 = moderately; 5 = extremely).

**Table 7:** Effect of the herb addition on the mean scores for the significant food-evoked emotions as rated by a group of older adults (*n* = 54) in a set of six thermally sterilized chicken pasta meals with different salt concentrations.

Herb Addition	Food-Evoked Emotions					
	Active	Aggressive	Bored	Calm	Happy	Wild
F (herbs added) ^1^	2.0 ± 1.0 a ^2^	1.5 ± 0.9 a	1.7 ± 0.9 a	2.6 ± 1.1 a	2.4 ± 1.1 a	1.4 ± 0.8 a
B (no herbs)	1.8 ± 1.0 b	1.3 ± 0.8 b	2.2 ± 1.2 b	2.8 ± 1.1 b	2.3 ± 1.0 b	1.2 ± 0.7 b
*p*-value	0.003	*p* = 0.006	<0.0001	*p* = 0.002	*p* = 0.002	*p* = 0.02

^1^ For each type of meal, meals with herbs and seasoning added are represented as F; meals with no herbs nor seasoning added are represented as B. The data for the mean estimation were collapsed along salt level. ^2^ Results are presented as the means scores ± S.D. Different letters within a column (a, b) indicate that the tested emotion mean value was different among the meals (*p* < 0.05), as determined by using Tukey’s HSD on a 5-point scale (1 = not at all; 3 = moderately; 5 = extremely).

**Table 8:** Mean values for the different indexes/scales determined based on the responses of 49 participants to the food choices questionnaire.

Scale/Index	Statistic							Range of Possible Scores
	1st Quartile	Median	3rd Quartile	Mean	S.D ^1^	Minimum	Maximum	
General health interest (GHI)	30.0	37.0	42.0	36.4	8.3	19.0	54.0	8–56 [36]
Food technology neophobia (FTN)	49.0	53.0	57.0	53.5	7.1	39.0	70.0	13–91 [41]
Inflexibility index (IFI)	23.0	30.0	36.0	30.7	10.2	15.0	56.0	12–84 [40]
Discretionary salt use	9.0	11.0	12.0	10.5	2.7	4.0	16.0	4–28 [34]
Personal norms	10.0	13.0	15.0	12.5	3.1	4.0	19.0	4–28 [34]
Awareness of the positive consequences of salt consumption	17.0	18.0	20.0	17.8	2.4	9.0	22.0	10–70 [34]
Awareness of the negative consequences of salt consumption	19.0	20.0	20.0	20.0	2.3	14.0	25.0	10–70 [34]

^1^ SD: standard deviation.

## Data Availability

Data are contained within the article.
